# Nanoparticle-Based Adjuvants and Delivery Systems for Modern Vaccines

**DOI:** 10.3390/vaccines11071172

**Published:** 2023-06-29

**Authors:** Brankica Filipić, Ivana Pantelić, Ines Nikolić, Dragomira Majhen, Zorica Stojić-Vukanić, Snežana Savić, Danina Krajišnik

**Affiliations:** 1 Department of Microbiology and Immunology, University of Belgrade-Faculty of Pharmacy, 11000 Belgrade, Serbia; brankica.filipic@pharmacy.bg.ac.rs (B.F.); zorica.stojic-vukanic@pharmacy.bg.ac.rs (Z.S.-V.); 2 Department of Pharmaceutical Technology and Cosmetology, University of Belgrade-Faculty of Pharmacy, 11000 Belgrade, Serbia; ines.nikolic@pharmacy.bg.ac.rs (I.N.); snezana.savic@pharmacy.bg.ac.rs (S.S.); danina.krajisnik@pharmacy.bg.ac.rs (D.K.); 3 Section of Pharmaceutical Sciences, University of Geneva, 1206 Geneva, Switzerland; 4 Division of Molecular Biology, Ruđer Bošković Institute, 10000 Zagreb, Croatia; dragomira.majhen@irb.hr

**Keywords:** nanoadjuvants, nanoemulsions, viral-vectored system, virus-like particles

## Abstract

Ever since the development of the first vaccine, vaccination has had the great impact on global health, leading to the decrease in the burden of numerous infectious diseases. However, there is a constant need to improve existing vaccines and develop new vaccination strategies and vaccine platforms that induce a broader immune response compared to traditional vaccines. Modern vaccines tend to rely on certain nanotechnology platforms but are still expected to be readily available and easy for large-scale manufacturing and to induce a durable immune response. In this review, we present an overview of the most promising nanoadjuvants and nanoparticulate delivery systems and discuss their benefits from tehchnological and immunological standpoints as well as their objective drawbacks and possible side effects. The presented nano alums, silica and clay nanoparticles, nanoemulsions, adenoviral-vectored systems, adeno-associated viral vectors, vesicular stomatitis viral vectors, lentiviral vectors, virus-like particles (including bacteriophage-based ones) and virosomes indicate that vaccine developers can now choose different adjuvants and/or delivery systems as per the requirement, specific to combatting different infectious diseases.

## 1. Introduction

The severe acute respiratory syndrome coronavirus 2 (SARS-CoV-2) pandemic served as a global reminder of how important the vaccinology field is. Modern vaccines are expected to combat a range of infectious and non-infectious diseases in a safe and efficacious manner while being readily available to developing countries as well [1]. However, conventional vaccine formulations generally favor humoral immunity, i.e., antibody-mediated responses, but fail to elicit strong cellular immune responses [2]. After decades of research in the nanotechnology field, nanostructured vaccine formulations (nanovaccines) were able to support different (alternative) routes of administration and offer facilitated uptake by the targeted cells while even modulating the immune response [3]. Moreover, lipid-based nanoparticles’ successful translation from other drug targets (e.g., patisiran story) to the vaccinology field (namely, mRNA coronavirus disease 2019 (COVID-19) vaccines) implied the potential of other carrier platforms to be correspondingly successfully used in vaccines [4,5].

This review aims to offer a summarized description of nanotechnology advances that resulted in readily used nanoadjuvants and/or nanocarriers able to enhance a nanovaccine’s immunization effect. According to the authors’ opinions, certain inorganic nanoadjuvants, nanoemulsions, viral-vectored systems, virus-like particles and virosomes were focused on as nanoplatforms of special interest for the potentially successful translation to emerging targets. Novel insights into the design of the abovementioned nanoadjuvants and nanocarriers are given, along with distinct strengths and weaknesses, due to the fact that their inherent composition may often be subjected to certain modifications in order to better suit the intended purpose.

A critical search of the publications was performed through PubMed (Rockwille Pike Bethesda, USA), Scopus (London, UK), Web of Science (Philadelphia, PA, USA) and institutional libraries (namely, Serbian Library Consortium for Coordinated Acquisition—KoBSON, Belgrade, Serbia and National and University Library in Zagreb—NSK, Zagreb, Croatia). Both review and original scientific papers were considered, published between January 2000 and January 2023 (papers published before 2000 were considered, when necessary). Initial, broader search served to identify information and knowledge gaps in already published review papers dealing with nanosized adjuvants and nanostructured vaccine formulations. Although the presented paper is not a systematic review, a description of the mode of data collection and selection may facilitate other authors in critically assessing and further updating given information.

## 2. Nanoadjuvants

Adjuvants have been widely used within vaccine formulations, for decades even, to improve the immune response to target vaccine antigens. Recently, so-called nanoadjuvants have tended to prevail in modern vaccine formulation strategies [2,6,7]. For successful vaccine development, it is of high importance to select an appropriate adjuvant and, subsequently, a suitable adjuvant-antigen combination in order to obtain stable, immunogenic and safe vaccines [8]. A common classification of vaccine adjuvants considers that an adjuvant can act either as an immunostimulant (directly interacting with the immune system, increasing the response to antigens; e.g., bacterial toxins, cytokines, saponins, Toll-like receptor (TLR) ligands) or as a vehicle (which introduces the antigen to the immune system, providing a controlled delivery of antigens or immunostimulants; e.g., aluminium salts, nanoemulsions, liposomes, virosomes, etc.). A combination of an immunostimulant and a vehicle is marked as an adjuvant system [9].

Despite the widespread use of adjuvants, the mechanisms of action of the currently available adjuvants in licensed vaccines, at least in humans, are not yet well understood. Nevertheless, today, it is known that innate immune cells, especially dendritic cells (DCs), which are the major antigen-presenting cells (APCs), can sense pathogen- and damage-associated molecular patterns (PAMP and DAMP, respectively) by different receptors (such as TLR, NOD- and RIG-like receptors). Many of these molecules, which could be part of adjuvants or components released from injured or dying cells on the site of the vaccine injection, induce inflammation, activate DCs and induce adaptive immunity mediated by vaccine antigen-specific T and B lymphocytes [10,11,12,13,14]. Briefly, during the recognition of vaccine antigens presented on DCs by the CD4+ T helper (Th) cell, DC-derived cytokines and costimulatory molecules on their cell surface influence Th cell differentiation towards three major subsets called Th1, Th2 and Th17 [10]. These Th cell subsets secrete different sets of cytokines and function in the host defense against distinct types of infectious pathogens. Th1 cells secrete interferon (IFN)-γ, and this type of immunity is associated with an increased macrophage effector function (killing of phagocytosed microbes) and protection against intracellular pathogens (bacteria and viruses). In mice, IFN-γ induces the production of IgG antibody isotype (IgG2a) by effector B cells, with the ability to neutralize pathogens, enhance phagocytosis of opsonized microbes and activate complement. However, there is no evidence that in humans generation of IgG antibody isotypes (IgG1/3) with same functions involves IFN-γ. Cytokines produced by DCs and Th1 cells during CD8+ T cell activation also promote the proliferation of CD8+ T cells and their differentiation into cytotoxic T cells, which eliminate intracellular pathogens by killing infected host cells. Th2 cells generally induce an antibody-predominant response whose physiological function is a defense against extracellular microbes. IL-4, the signature cytokine of the Th2 subset, stimulates B cells to enhance the production of IgG antibodies that can neutralize pathogens (IgG4 in humans and IgG1 in mice) but do not bind to phagocyte Fc receptors or activate the complement. Th17 cells play a critical role in host defense against a variety of bacteria and fungi that can survive outside cells, primarily by producing the IL-17 cytokine, but data regarding Th17-inducing adjuvants are still scarce. Having all of this in mind, a major goal of the next-generation vaccines is the selective use of adjuvants and delivery systems to enhance optimal immune responses in humans [11,12,13,14].

In the era of modern vaccinology, research has been refocused towards novel adjuvants, and antigen administration is usually performed by applying nanoparticulated delivery systems, rendering more effective vaccines [15,16]. Therefore, in this section, novel inorganic nanoadjuvants and nanoemulsions as lipid-based adjuvants/adjuvant systems will be specially addressed. Their main immunological properties and clinical application are summarized in Table 1.

### 2.1. Inorganic Nanomaterials as Vaccine Adjuvants and/or Carriers

#### 2.1.1. Conventional Alum Adjuvants

**Figure 1:**
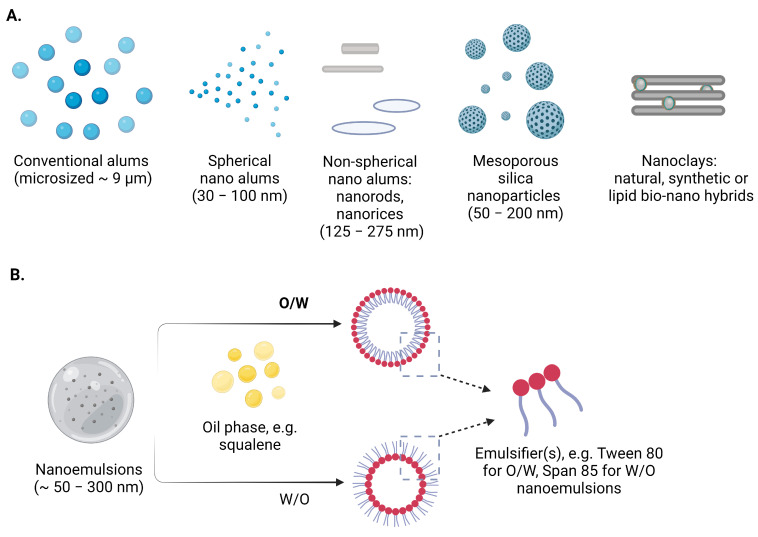
Schematic representation of the selected (**A**) inorganic nanomaterials (micro- to nanosized) and (**B**) nanoemulsions as adjuvants and/or carriers in contemporary vaccine formulations. Created with BioRender.com.

Aluminum-containing adjuvants (Alums) have historically served as immunostimulants in vaccines and continue to be the most widely used adjuvants generally regarded as safe [17]. Several aluminium compounds (aluminum hydroxide, aluminum phosphate and amorphous aluminum hydroxyphosphate sulfate) are used in the licensed vaccines (for intramuscular or subcutaneous applications) [18]; however, their distinctive physicochemical properties could have important implications for their immunomodulatory effects [19] (Figure 1).

The primary nanoparticles of aluminium adjuvants (aluminum hydroxide and aluminum phosphate) are fibers with an average size of 4.5 × 2.2 × 10 nm that form loosely connected porous aggregates. These aggregates present the functioning units in vaccines, and their size varies between 1 and about 20 μm [20] related to the adjuvant, particle size measurement method, and testing conditions. Along with the particle size, aluminium adjuvant’s surface charges could differ notably, namely, the isoelectric point (IEP) of these adjuvants varies from 4.6 to 11.1 depending on the salt [21,22], resulting in different charges in the physiological environment, which can be of great significance for the interaction with the antigen [19,23,24].

The formulation of vaccines with aluminium salts requires the careful selection of excipients such as buffers, tonicity-adjusting agents, surfactants and other stabilizers [24,25,26]. The thermostability of vaccines containing aluminium adjuvants is of great importance, since exposure to both high and low temperatures (particularly freezing) can affect the stability of vaccines and potentially cause irreversible coagulation/agglomeration [22,27]. Although several technologies have been proposed to overcome sensitivity to freezing conditions [28,29,30,31], this remains one of the main drawbacks of these adjuvants. Aluminium-containing adjuvants can potentiate the immune response by: (i) forming a depot at the site of injection, which is associated with slow release of antigen, (ii) targeting antigens to APCs and enhancing their uptake, and (iii) direct stimulation of NOD-like re-ceptor protein 3 (NLRP3) and formation of the NLRP3 inflammasome, which generate the biologically active form of the inflammatory cytokines interleukin (IL)-1β and IL-18 [22,32,33,34,35]. Traditionally, alums enhance Th2 responses to protein antigens and promote antibody responses but have minimal effect on cell-mediated immunity, especially in mice. However, mixed Th1/Th2 immune responses can be primed in humans when aluminium-based adjuvants are injected intramuscularly [33,34].

It has been apparent for some time now that satisfactory immunization outcomes are highly dependent on both objective side effects and the ones subjectively perceived for certain vaccines’ components (e.g., effects emphasized by the patient’s apprehensions). Moreover, it needs to be accepted that enhancing immunity inevitably leads to some undesired effects [36]. Alum adjuvants are generally deemed safe, and usually held responsible for local adverse reactions such as pain/tenderness at the injection site, accompanied by various degrees of inflammation, erythema, subcutaneous nodules and/or granuloma [37]. Additionally, in susceptible patients, alum adjuvants may trigger allergic reactions of various intensities, which appear to be alum concentration-dependent. However, intramuscularly administered alum vaccines may lead to more serious macrophagic myofasciitis-like local tissue impairments [38].

#### 2.1.2. Nano Alum Adjuvants

Inorganic materials facilitate sustained and targeted release of antigens, enhance immunogenicity, and elicit a specific immune response. Over the past decade, inorganic materials have been intensively studied as vaccine adjuvants on the basisof nanoscale synthesis, and control of structural and functional properties. Moreover, it is possible to control their structural, optical, electrical or magnetic properties by engineering design, which allows the choise of the most suitable adjuvant formulations by means of in vitro and in vivo investigations [39,40,41].

It was confirmed by Sun and co-workers [41] that changes in alumina nanoparticles related to hydroxyl content, their shape and crystallinity had a significant influence in the activation of the NLRP3 inflammosome and improvement of antigen-specific immune responses. Particle size reduction of the conventional alum adjuvant to a nanometer scale (~112 nm) compared to mi-croscopic particles (~9 μm) elicited a stronger antigen-specific antibody response, which was likely related to their ability to effectively facilitate the uptake of adsorbed antigens by APCs [32]. A stronger adjuvant activity of insoluble aluminium oxyhydroxide nanoparticles (~30–100 nm) compared to microparticles (X50, 9.43 μm) was attributed to their stronger ability to stimulate uric acid production and consequently indirectly activate the inflammasome [42]. An evaluation of the specific immune response after the administration of aluminum hydroxide nanoparticles containingthe EsxV antigen of *Mycobacterium tuberculosis* (~200 nm) demonstrated strong activity in stimulating a Th1-type cellular immune response making them suitable adjuvant against *M. tuberculosis* infection [43].

Recently, researchers synthesized a series of non-spherical Alum nanoadjuvants (so-called nanorods or nanorices), showing satisfactory suspension stability upon model antigens’ adsorption [6,44]. These nanoadjuvants are attributed with different aspect ratios but are generally characterized by hydrodynamic sizes ranging from 125 to 275 nm. Improved suspension stability indices were demonstrated both in water, saline and buffers, compared to commercially available Alums.

The effects of the surface coating of aluminium adjuvants were also investigated. Phospholipid bilayer-coated aluminium nanoparticles (PLANs; 50 nm in size) are formed via chemisorption and prepared by reverse ethanol injection–lyophilization. The anhydrous antigen-loaded PLANs showed satisfactory stability under the controlled-temperature-chain instead of the integrated cold-chain for distribution. Additionally, the coated adjuvants may stimulate powerful antigen-specific humoral and cellular immune responses with less local inflammation because they were more readily taken up by APCs [45]. Of course, the fact that higher positive zeta potentials of nanoadjuvants favor the resulting suspension stability was considered during Alum nanoparticles’ engineering [46]. The cationic surface functionalization of aluminum oxyhydroxide nanorods (ALNRs) showed that NH2-functionalized ALNRs had higher levels of cellular uptake, lysosomal damage, oxidative stress, and NLRP3 inflammasome activation in cells than untreated and SO3H-functionalized ALNRs [47]. Similarly, Ren et al. [48] found that coating conventional Alums with polyethyleneimine could boost antigen cross-presentation by DCs to CD8+ cytotoxic T cells in a clinically relevant manner.

The findings available so far suggest that nano alums may trigger the abovementioned adverse reactions faster than the conventional alum adjuvants. On one hand, this may be regarded as another benefit of nano alums, considering the fact that certain side effects may be observed early by the healthcare provider upon vaccine administration. On the other hand, some researchers fear that nano alums may provoke more serious subcutaneous granuloma and other local inflammatory reactions [34]. Undoubtedly, the safety of nano-sized adjuvants will be continually scrutinized by both the researchers and regulatory authorities.

#### 2.1.3. Silica Nanoparticles

The intrinsic structural characteristics of designed mesoporous silica nanoparticles (MSNs) (with an average size of 50 to 200 nm) such as the tailorable mesoporous structure, high specific surface area, large pore volume, low density, good biocompatibility, thermal and chemical stability and easy chemical functionalization, contributed to their various biomedical applications [45,49,50]. The in vivo behavior of MSNs is highly dependent on their preparation methods, particle sizes, geometries, surface chemistry, dosing regimens, and even routes of administration, as shown by systematic biosafety assessments [51,52,53]. In animal models, the use of MSN as an immune adjuvant has been shown to effectively enhance both cellular and humoral immunity [54,55]. As a representative, bovine serum albumin (BSA) encapsulated/adsorbed SBA-15 nanostructured silica (a mesopore diameter of ~10 nm) had the ability to increase the immunogenicity and stimulate mutually Th1 and Th2 immune responses via intramuscular or oral administration in mice [56,57], while the intraperitoneal injection of ovalbumin and amorphous silica nanoparticles (diameter of 33 nm) exerts an adjuvant effect for Th1, Th2 and Th17 immune responses [58]. Additionally, SBA-15 was demonstrated to be an efficient carrier for the hepatitis B surface antigen (HBsAg) as well as diphtheria anatoxin for oral and/or subcutaneous vaccination [56,57,58,59,60,61]. Therefore, MSNs could be a valuable adjuvant for divalent and trivalent vaccine formulations. In the future, significant progress is expected in the rational design and tailored functionalization of MSN-based protein delivery systems, as well as in the evaluation of biocompatibility, stability, efficacy, and biological [62,63].

#### 2.1.4. Clay Nanoparticles

Clay nanoparticles (or nanoclays), such as layered double hydroxides and hectorite nanoparticles, exhibited strong adjuvant activity leading to immune responses significantly potent than those obtained by adjuvants which are available on the market (such as Quil-A^®^ and alum) [64]. Although natural clays are also investigated, the majority of research groups work with synthetic nanoclays [65]. Surface modifications through clay–organic interactions resulted in formation of hybrid organic–inorganic materials, where the added organic functionality leads to complementary qualities to those intrinsic to the silicate substrate [66]. Wicklein et al. [67] synthetized lipid-based bio-nano hybrids as carriers of viral particles in vaccines against influenza A, using sepiolite as a biocompatibile nanoclay modified with phosphatidylcholine. This clay-lipid formulated influenza A vaccine exhibited increased thermal stability in functional tests at elevated temperatures up to 48 °C and showed high immunogenicity in mice with strong induction of specific antibodies associated with a Th1 cell cytokine profile [67].

A variety of other representatives of inorganic nanomaterials such as gold and carbon nanoparticles, oxides and hydroxides (e.g., zinc oxide, iron oxide and iron hydroxide), quantum dots, nanodiamonds, etc. have been investigated as potential vaccine adjuvants [40,68]. A somewhat broader range of metal-based nanomaterials are being evaluated as potential adjuvants in nanovaccines for cancer immunotherapy [69].

## 3. Nanoemulsions

Nanoscale emulsions (nanoemulsions) represent heterogeneous systems consisting of two liquids of opposite polarity (therefore, imisscible), where one of them is dispersed in the other one, forming something very small—usually 50–300 nm in diameter. These dispersions can be water-in-oil (W/O) or oil-in-water (O/W) (Figure 1). Their physical stability is ensured through the appropriate selection of components and the preparation technique [70]. Nanoemulsions are characterized by numerous properties that are attractive for biomedical applications. These colloidal systems have been explored as carriers/vehicles for hydrophobic compounds, covering various routes of administration: parenteral, dermal, oral, ocular and nasal [71,72,73,74]. In the context of vaccine development, nanoemulsions have been used in both human and animal vaccines as lipid-based adjuvants [74]. In addition to their role in the presentation of the antigens to the immune system, emulsions promote slow antigen release and protection from swift elimination [75].

In approved vaccines, nanoemulsions were presented in the 1990s, when the pharmaceutical company Novartis, Basel, Switzerland registered the first emulsion-based adjuvant in Europe [76]. Indeed, from a historical point of view, the adjuvant effect of emulsions (precisely, paraffin oil in a W/O emulsion) was known more than eighty years ago, having been demonstrated by Freund. However, these first attempts to boost the immune response by applying Freund’s complete adjuvant (W/O emulsion with thermally killed mycobacteria) and, afterwards, Freund’s incomplete adjuvant (W/O emulsion without bacterial cells) were discouraged, as such systems proved to be too reactogenic for general applications [76,77]. Even so, insights into the adjuvant efficacy of (nano)emulsions were not dismissed. The subsequent experience and improvement of the raw materials used for nanoemulsion preparation segregated essential physical properties for an efficient and safe nanoemulsion-based adjuvant, such as: the droplet size and surface properties, O/W ratio and rheological behavior [26,78,79,80].

**Figure 2:**
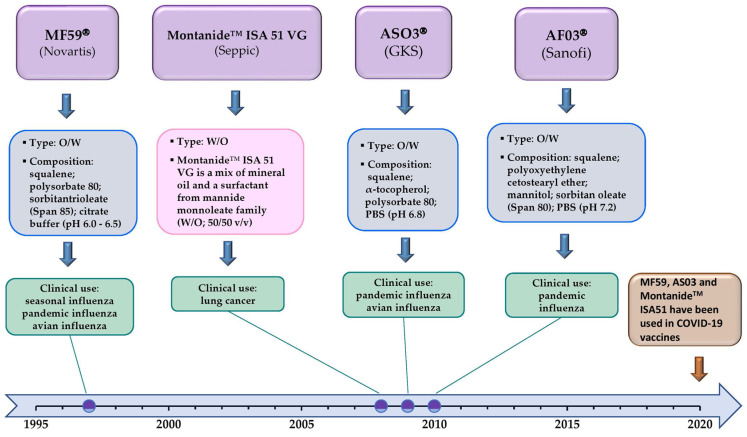
Timeline of nanoemulsion-based adjuvants’ research and application.

With a view to optimize the safety/immunogenicity ratio, it has been established that tolerability can be ameliorated by replacing W/O with O/W nanoemulsions. A reduction in the oil concentration is followed by a decrease in the droplet size and viscosity. Such shift enabled an easier injection and eliminated the tendency of the emulsion to stay at the injection site, causing reactogenity [22,81]. In addition, O/W emulsions offer the advantage of the simple mixing of aquous antigen solutions with preformed emulsion. Therefore, antigens and adjuvants may be stored separately and combined afterwards, just before the administration [82,83]. The investigation of other types of oil acceptable for adjuvant nanoemulsion formulations has also led to significant progress: mineral oil has been replaced by squalene-based formulations (Figure 2), as it is fully metabolizable and less toxic [74,84,85].

One of the most widely used nanoemulsion-based adjuvants is MF59^®^ (created by Novartis in 1997), containing 5% *v*/*v* of squalene as the oil phase and showing good performances in influenza vaccines [86]. Through extensive in vitro cell assays, Seubert et al. demonstrated that MF59^®^ may increase immune cell migration to the injection site, promote monocyte differentiation towards DCs and consequently stimulate their maturation and antigen uptake while enhancing migration to the lymph nodes [87]. MF59 stimulates both Th1/Th2 cells, leading to humoral and cellular immune responses [18]. Another example of squalene-based nanoemulsion is the DETOX adjuvant system, developed by Ribi ImmunoChem Research, Hamilton, MO, USA (later acquired by Glaxo Smith Kline, London, UK (GSK)), containing a purified mycobacterium cell wall skeleton and monophospohoryl lipid A (as an immunostimulant; TLR4 ligand) dissolved in squalene. It is a specific adjuvant system used in a therapeutic melanoma vaccine called Melacine^®^ [85,88,89]. Good illustrations of nanoemulsion-based adjuvants with squalene are ASO3^®^ (containing alpha-tocopherol dissolved in squalene as an immunostimulant; developed by GSK) [90] and AFO3^®^ (with sorbitan oleate and polyoxyethilenecetostearyl ether as stabilizers; created by Sanofi Pasteur, Lyon, France) [82]. AS03 has a similar mechanism of action to that of MF59 [18]. Additionallly, in the study of Morel et al. [90], it was depicted that the ability of ASO3^®^ to induce an antigen-specific antibody response is likely linked to its capacity to activate innate immunity. The obtained results confirmed the good safety profile of ASO3^®^-containing vaccines. However, some findings imply that ASO3^®^ may be responsible for the reported narcolepsy cases after pediatric influenza vaccine administration [91]. On the other hand, the manufacturing peculiarity of AFO3^®^ is that, unlike for other submicron emulsions, the phase inversion temperature procedure—a low-energy emulsification technique—was applied [82]. This technique is basically driven by the inherent physicochemical properties of the ingredients, not requiring sophisticated and expensive equipment [70]. This approach has effectively minimized both the development timelines and costs in industrial settings [84]. In response to the H1N1 influenza outbreak in 2009, European regulatory authorities granted approval to three pandemic influenza vaccines containing O/W emulsions (MF59^®^, AS03^®^ and AF03^®^) as adjuvants [22]. Expectedly, the years that followed resulted in the new versions of W/O adjuvant nanoemulsions, among which the Seppic’s (Courbevoie, France) Montanide^®^ adjuvants (e.g., ISA 720, ISA 51), also squalene-based, can be underlined [92,93,94].

Unsurprisingly, the literature describes more examples of nanoemulsion-based adjuvants that are under investigation, such as CoVaccine HT^®^ and Stable emulsion^®^ (SE). CoVaccine HT^®^ represents a combination of a sucrose fatty acid sulfate ester and squalane in a nanoemulsion for a single-dose pandemic influenza vaccine that is able to induce efficient Th1-biased cellular and humoral immune responses [95]. The study recently published by Lai et al. [96] demonstrated that CoVaccine HT^®^ significantly contributes to robust immune responses against several SARS-CoV-2 variants.

Along with immunological behavior, physicochemical stability is one of the primary concerns for the nanoemulsion formulators. Both the physical instability (manifested either as droplet flocculation, Ostwald ripening, coalescence, creaming) and/or chemical degradation of nanoemulsion components jeopardize the adjuvant efficacy and safety. In addition, the factors related to potential freeze drying, which represents a common technique that promotes stability, facilitates transport and prolongs the product’s shelf-life, ought to be addressed [97,98]. These issues should be overcome by the careful selection of aqueous phase components, oils, stabilizers and their concentration and mutual ratio, but the preparation technique must not be neglected [70,99]. Apparently, the majority of the approved nanoemulsions-based adjuvants have been formulated with squalene—a naturally derived and metabolizable oil—due to the good human safety track record of MF59^®^ [82,100]. There has been some research covering the possibility of using other metabolizable oils, such as soybean, peanut or coconut oil. However, squalene over-performed their ability to provide nanoemulsions with an effective immune response and desirable physical properties [84,85,101]. Likewise, Tween 80^®^, as a well-established emulsifier, is applied as a stabilizer in most cases [84,102].

Last but not least, the COVID-19 vaccine development has also profited from the possibilities of lipid-based adjuvants. MF59, AS03, Freund’s adjuvant and Montanide ISA51 have been used in COVID-19 vaccines [103,104]. GSK has been sharing its AS03 adjuvant with COVID-19 vaccine developers, and VidPrevtyn Beta, a recombinant COVID-19 vaccine developed by Sanofi, contains the ASO3 adjuvant and is approved by the European Commission as a booster dose vaccine for preventing COVID-19 in people aged 18 years and older [105,106].

In addition, the lipid nanoparticles, as another lipid-based nanosystem, especially underlined in the context of COVID-19 vaccines [107], have also been identified as a potent immunostimulatory component of mRNA and protein subunit vaccines (eliciting IL-6 production that is essential for antibody responses) [108,109].

Numerous studies point out a complex interplay among the droplet/particle size, surfactant concentration and immunostimulant components that have a significant effect on the biological/immunological performances of vaccine adjuvants [110,111,112,113]. Over the past two decades, noteworthy progress in the development of novel vaccine adjuvants has been made. Still, adjuvant activity is a result of multiple factors, and an enhanced immune response obtained with one antigen cannot be taken as a rule and extrapolated to another antigen [75]. A deeper understanding of the relationship among formulation science and relevant physical, chemical and physiological challenges related to specific pathological conditions, routes of administration and patient populations (children, adults, elderly) is required for rational and knowledge-based adjuvant design. Consequently, more potent adjuvants and improved vaccination compliance are envisaged. Taken all together, following their intensive human use worldwide, the benefits and safety profiles of nanoemulsion-adjuvanted vaccines are now very well established [78].

## 4. Virus-Based Nanocarriers as Vaccine Delivery Systems

The use of nanocarriers originated from natural sources that have specific properties such as the ability to escape host immune response and to enter the host cell in order to deliver specific genetic material, has become an important tool in the vaccine field [14]. Good example of naturaly originated nanocarriers that may have these characteristics are viruses that are an important class of naturally occurring bioinspired nanosystems for vaccine delivery [114]. Viruses can be rendered non-infectious and non-replicable without losing their ability to enter host cells, and since viral proteins exhibit ideal properties such as biocompatibility, biodegradability and uniform size distribution, all these characteristics underscore and encourage the use of viruses as vaccine delivery systems [115]. Traditional bio-based nanoparticle delivery systems such as liposomes present several challenges such as: less specific target delivery, poor stability in biological fluids, difficulties in mass production and in vivo toxicity that can be overcome with viral nanoparticles [14]. In addition, adjuvants currently used in humans have been observed to enhance humoral immunity; however, virus-based nanocarriers have advantage comparing to traditional adjuvants as they can induce both humoral and broad spectrum of cellular immune responses [116,117]. Different viral vectors with desired genetic information inserted into viral genome are used as vehicles: adenoviral, adeno-associated viral, vesicular stomatitis viral, lentiviral vector, etc. These vaccine platforms can be made using replicating or non-replicating viral vectors. Moreoften, the viral vectors are modified to be nonreplicating, as it is considered that nonreplicating viral vectors are safer than replicating viral vectors and have reduced reactogenicity. On the other hand, nonreplicating viral vectors may have lower immunogenicity profile requiring higher dose or repeated doses, and that’s why in some cases the use of replicating viral vectors (e.g. vesicular stomatitis viral vector) is preferred [118,119].

**Figure 3:**
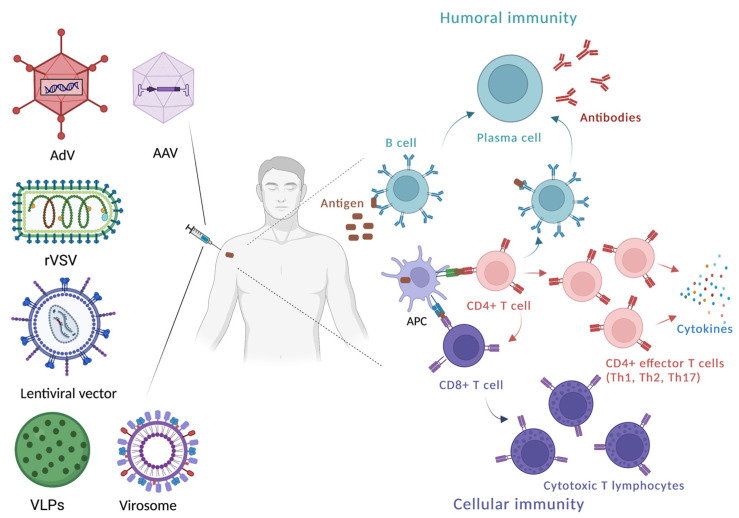
Different virus-based nanocarriers as vaccine delivery systems which function by activating cellular and humoral immunity to varying degrees. Apart from other presented virus-based nanocarriers, VLPs (including bacteriophage-based VLPs) and virosomes deliver antigens instead of a genetic material coding antigen structure. AdV—adeno-viral vector; AAV—adeno-associated viral vector; rVSV—recombinant vesicular stomatitis virus; VLPs—virus-like particles, APC—antigen-presenting cells. Created with BioRender.com.

Additionally, virus-derived features such as virus-like particles (VLPs) and virosomes are very attractive platforms for vaccine development as they are safe and stable, morphologically similar to the infectious virus with the ability to enter the host cell and induce both humoral and cellular immunity (Th1, Th2 and CD8+ T cell immune response) (Table 1) [120]. So, in this section, viral-vectored based delivery systems, as well as VLPs and virosomes, will be described in more detail as important virus-based bionanocarriers for vaccine delivery (Figure 3).

### 4.1. Viral-Vectored-Based Delivery Systems

Recombinant viral vectors have been shown to be profoundly effective vehicles for bringing foreign nucleic acids into target cells. Furthermore, infections naturally induce host immune reactions, which might be beneficial for vaccination purposes. Both of these features, joined by broad knowledge on techniques for manipulating the viral genome, make recombinant viral vectors appealing candidates for vaccine vector development. Recombinant viral vectors have been and are being examined as vaccines focusing on different viral, bacterial and protozoan microbes. They are especially utilized in disease areas where traditional vaccination approaches have been demonstrated to be ineffectual, troublesome or technically impossible. So far, viral vectors vaccines have been used as an approach in treating different infectious diseases including COVID-19, human immunodeficiency virus (HIV), Malaria, Ebola and others. Likewise, different viruses such as adenovirus, adeno-associated virus, vesicular stomatitis virus or lentivirus have been used for constructing recombinant viral vectors [118].

#### 4.1.1. Adenoviral Vector-Based Delivery System

Adenoviral vectors possess the ability to trigger strong immune responses on cellular, humoral, and mucosal levels, rendering them highly appealing for the development of vaccines.Compared to traditional vaccines, a diverse range of antigens can be incorporated into adenovirus vaccines to prompt an immune response that targets a wide array of pathogens while also acting as an adjuvant for an antigen-encoding vector. 

By employing uncommon serotypes of both human and non-human origin, the issue of pre-existing immunity is effectively overcome,, while booster regimens with alternative adenoviral vectors have been shown to be safe and immunogenicVaccination strategies utilizing recombinant adenovirus-based vectors show great potential, especially in the field of infectious diseases where cellular immune responses are crucial for protection. Adenovirus-based vectors indeed stimulate robust and durable cellular responses, particularly in CD8+ T cells. [121].

So far, different adenovirus serotypes have been employed as vaccine vectors, the most prominent being human adenovirus type 5 (HAdV-C5), human adenovirus type 26 (HadV-D26) and chimpanzee adenovirus, ChAdOx1. Due to the fact that it has the capability to trigger immune responses involving both humoral and cell-mediated components , HAdV-C5 has been the most common choice for preclinical studies of adenovirus-based vector vaccines. In one of the first HAdV-C5 based vector vaccine studies, an efficacy assessment of a cell-mediated immunity to the HIV-1 vaccine (the Step Study) was performed, and, surprisingly, an increase in the number of HIV-1 infections in male recipients of the vaccine was noted [122]. Even though this could have been considered as a failure, the development of HAdV-C5-based vaccine vectors continued and peaked during the COVID-19 pandemic. A single vaccination with the replication defect HAdV-C5 encoding the spike protein of SARS-CoV-2 (Ad5-nCoV) was shown to completely protect mice from infection of the upper and lower respiratory tract with mouse-adapted SARS-CoV-2. This suggests that mucosal vaccination may provide the desired protective effect; thus, this route of administration requires further investigation in human clinical trials [123]. Later on, the Ad5-nCoV entered the clinical trials [124,125], and it was approved for use in 2021 in China, Hungary, Mexico and Pakistan.

Due to itslow occurrence in human populations and its ability to elicit a favorable immune response to inserted transgenes, HAdV-D26 has emerged as a promising framework for the development of vaccine vectors. Consequently, it has been the subject of numerous ongoing and completed clinical studies. HAdV-D26-based vaccine vectors have been assessed as potential interventions for diseases associated with HIV, respiratory syncytial virus (RSV), Ebola virus, Zika virus and SARS-CoV-2. Very recently, the European Medicines Agency (EMA) approved two HAdV-D26-based vector vaccines, namely, Ad26.ZEBOV against Ebola [126] and Ad26.COV2.S against COVID-19 [127]. The simian adenovirus-based vaccine vector ChAdOx1 against COVID-19 [128] has also been approved by regulatory agencies.

Rare occurrences of thrombocytopenia have been documented following the administration of adenoviral vector-based COVID-19 vaccines. A recently published study revealed that three adenoviruses utilized as vaccination vectors against SARS-CoV-2, namely HAdV-C5, HAdV-D26, and ChAdOx1, exhibit binding affinity towards platelet factor 4 (PF4), a protein implicated in the development of heparin-induced thrombocytopenia. The findings presented by these researchers suggest that stable complexes formed between PF4 and clinically relevant adenoviruses may play a significant role in elucidating the mechanisms underlying thrombosis with thrombocytopenia syndrome. [129]. These results underscore the necessity for further investigations into adenovirus-based vaccine vectors.

#### 4.1.2. Adeno-Associated Viral Vector as a Delivery System for Vaccines

Adeno-associated virus (AAV) vectors have been extensively investigated as gene therapy vectors and have only recently gained attention in the field of vaccination. However, with the discovery that AAV can elicit a robust humoral and cellular immune response against the glycoprotein B of herpes simplex virus (HSV) type 1 [130], there has been a growing interest in exploring the application of AAV vectors for vaccination. Numerous studies have since delved into the utilization of AAV vectors for this purpose.Thus, it has been shown that a single injection of an AAV vector induced a potent and long-lasting antibody response without the need for an adjuvant [131,132]. Even though in some cases, compared to other vaccination strategies, AAV vectors have demonstrated the ability to generate a higher or longer-lasting antibody response. Additionally, they are regarded as having a low immunogenic profile in comparison to other viral vectors.. In addition to the low immunogenicity, AAV vectors suffer also from a very limited insertion space, namely, only 4.8 kb. However, AAV vectors are employed as vaccination vectors for delivering antigens. They facilitate the production of foreign proteins with a structure and arrangement that closely resemble those of natural pathogens [133], or as vehicles for delivering monoclonal antibodies and derivatives of immunoglobulins as a means of passive immunizations [134].

#### 4.1.3. Vesicular Stomatitis Viral Vector for Vaccines

For more than a decade, Vesicular stomatitis virus (VSV) has been acknowledged as a robust vaccine vector for infectious diseases. Recent clinical trials examining the VSV-EBOV vaccine candidate against the Ebola virus (EBOV) have contributed a substantial amount of clinical data, indicating that VSV holds great potential as an ideal vaccine candidate for epidemic pathogens. [135]. The safety and immunogenicity of the VSV-based recombinant viral vector (rVSV) have been demonstrated in pre-clinical and clinical trials against Ebola, HIV and SARS-CoV-2. An extensive pre-clinical and clinical evaluation of the VSV-based Ebola vaccine demonstrated remarkable safety and efficacy, which ultimately led to the U.S. Food and Drug Administration (FDA) licence (also known by its brand name, Ervebo, Merck & Co., Rahway, NJ, USA) for use in humans against EBOV infection [136]. A recent study has demonstrated that a replicating VSV-SARS-CoV-2 vaccine triggers the production of potent neutralizing antibodies at high levels. This finding provides strong support for advancing the development of VSV-SARS-CoV-2 as an attenuated, replication-competent vaccine against SARS-CoV-2. [137].

#### 4.1.4. Lentiviral Vector for Vaccines

Lentiviral vectors have become attractive as vaccine vectors in many preclinical animal models of [138] infection and oncology because of their high efficiency in transducing DCs and inducing long-term humoral and CD8+ immunity, as well as their potent protection. Recently, clear prophylactic effects of lentivirus-based vaccinations against SARS-CoV-2 have been described, indicating that intranasal vaccination is an effective approach against COVID-19. Namely, an intranasal strategy triggered a localized upper airway immune response that conferred disease protection in COVID-19 mice and hamster models [139]. Lentivirus vaccines have been tested in multiple preclinical studies in mice and rhesus monkeys, including against HIV, Zika and malaria, and have demonstrated high immunogenicity and sustained protective immune responses [140,141,142].

### 4.2. Virus-like Particles and Virosomes

#### 4.2.1. Virus-like Particles (VLPs)

Virus-like particles (VLPs) are empty (without viral genetic material), non-replicating and non-infectious structures that have ability to self-assemble [143]. VLPs are nanomaterials, and the expression and self-assembly of the viral structural proteins can take place in various expression systems (including mammalian cell lines, plants, insects, yeasts and bacteria), after which the VLPs self-assemble into the structure similar to original virus, but without pathogenic potential [144,145,146]. First VLPs derived from Hepatitis B virus were introduced in 1968, after which different types of VLPs have been developed and with history of more than fifty years VLP-based vaccines are considered as safe [147,148].

The main building unit of VLPs is surface protein that is antigen of interest displayed in high levels after self-assembling process [149]. After phagocytosis, these antigens may be presented by DCs leading consequently to the induction of both humoral and cellular immune responses [117,150]. The most common VLPs size is within the range of 20~200 nm in diameter, which is optimal for particles to be recognized by DCs and other APCs including B cells, which process and present peptides of interest to T cells in lymph nodes and stimulate T cell-dependent antibody immune responses [144,151,152]. However, as VLPs may display multimeric epitopes, they can promote the cross-linking of antigen receptors on B cells, which can be strong enough for activating them and also induce the antibodies production but without the T cell help (T cell-independent antibody immune response) (Figure 3). Therefore, in most cases, adjuvants are not needed in VLP-based vaccines, but the use of an adjuvant within the VLP-based vaccine formulation may improve their immunogenicity [153].

Hundreds of VLPs are in different stages of preclinical and clinical studies for numerous infectious diseases, including HIV, influenza, hepatitis B, hepatitis E, malaria, Ebola, COVID-19, Zika virus, Dengue and foot and mouth disease, among others [154]. Several VLPs vaccines are licensed and are already in use, such as Engerix-BR (GSK) and Recombivax HBR (Merck & Co) against hepatitis B virus, Gardasil (Merck & Co) and Cervarix (GSK) against human papilloma viruses (HPV), Hecolin (Xiamen Innovax Biotech Co., Xiamen, China) against hepatitis E virus [155] and MosquirixTM (GSK) against malaria [156]. VLPs are classified as subunit vaccines, as they are made of non-infectious viral components.

#### Bacteriophage-Based VLPs

Bacteriophages or phages are viruses that infect and replicate within bacteria. VLPs from a variety of RNA bacteriophages, particularly VLPs derived from Leviviridae, are the most studied bacteriophage-based VLPs with the great potential for being used as vaccine platforms for the antigen delivery [157]. Bacteriophages belonging to the family Leviviridae possess positive-sense single-stranded RNA (ssRNA) packed inside an icosahedral protein shell made of 178 copies of coat protein (CP), with the diameter of about 30 nm [158,159]. The recombinant expression of ssRNA phage CPs in bacteria or yeasts most often result in the efficient assembly of VLPs [159]. Bacteriphage-based VLPs can display wide range of different antigens, and there are two main mechanisms for these antigens to be produced: a) genetic fusion with CPs or b) chemical conjugation to the surface of VLPs [160]. Several bacteriophage-based VLPs have been created using phage CPs of PP7 (infects *Pseudomonas aeruginosa*), Qβ (infects *Escherichia coli*), MS2 (infects *Escherichia coli*) and AP205 (infects *Acinetobacter* bacteria) bacteriophages [159,160]. Some examples of bacteriophage-based VLPs that are in different stages of clinical studies are: the PP7-based HPV vaccine [161], Qβ-based COVID-19 vaccine [162], MS2-based foot and mouth disease vaccine and HPV vaccines [157,163] and AP205-based flu and COVID-19 vaccines [164,165], but there is no approved bacteriophage-based vaccine from EMA or FDA yet.

The size (about 30 nm) of bacteriophage-based VLPs is important, as it allows for the direct dranage of VLPs to the lymph nodes and optimal B-cell activation. Moreover, the important advantages are that both their surfaces and their interior contents can be modified, which enables that VLPs can display different epitopes on their surface or interior of the VLPs can be packed with TLR ligands, such as RNA or DNA sequences which can activate TLR7/8 and TLR9, respectively [166].

#### 4.2.2. Virosomes

Other delivery systems, also classified as subunit vaccines, similar to VLPs are virosomes. Virosomes, spherical, unilamellar vesicles (60–200 nm) comprising lipid nanomaterials, emerged as FDA-approved nanocarriers reconstituted of viral envelopes phospholipids with a removed nucleocapsid. Virosomes hold promising bioinspiration and biomimetic potency against viral infections [167]. The virosomes have a mono- or bilayer phospholipid envelope to which components/antigens originating from viruses or other pathogens can be attached or inserted and are preffered to VLPs because of the protein-based nature of VLPs [117]. The important advantage of virosomes is their ability to bind to the target cells through membrane fusion proteins retained from the native virus, which forms the basis for their enhanced immunogenicity. The virosomal surface contains antigen molecules that interact with receptors on B lymphocytes, which triggers a strong antibody response. Additionaly, virosomes efficiently interact with antigen-presenting cells, such as DCs, leading to the activation of the T lymphocites-mediated immune response [168] (Figure 3). Virosomes have shown promising results in vaccine development against various diseases including influenza and hepatitis A, for which the virosome-based formulations Inflexal^®^ V and Epaxal^®^ have been licenced, respectively. However, there is ongoing research to develop a potent vaccine against HIV-1, HPV and SARS-CoV-2 [167].

## 5. Conclusions

The application of nanotechnologies has led to great progress in the field of vaccine research and has become an important pillar of vaccinology. The great growth in this field enabled the development of new nano-sized vaccines and the design of nanoadjuvants and nanoparticulate delivery systems that are more reliable and/or effective and that can be applied in new medical treatments. The current findings imply that nanostructured vaccine formulations provide certain advantages in the elicited immune response and are suitable for a variety of administration routes and dose regimens. Some formulations benefit from the inclusion of a single nanoadjuvant, resulting in an improvement of the overall vaccination effect. Apart from most adjuvants currently used in humans, many new adjuvants (such as liposomes) or delivery systems (e.g., viral-vectored vaccines) elicit both humoral and specific T-cell responses. So far, certain inorganic nanoadjuvants (Alum-based, MSNs and metal-based nanomaterials), nanoemulsions (e.g., MF59^®^, ASO3^®^, AFO3^®^), viral-vectored systems, virus-like particles and virosomes have been of special interest for the potentially successful translation to emerging targets to deal with challenging diseases such as HIV infection, malaria, etc. All of these efforts enabled vaccine developers to choose different adjuvants and/or delivery systems as per the requirement, which was extremely important for the development of COVID-19 vaccines. It is expected that intense multidisciplinary research in the fields of nanomaterials, immunology, bacteriology, virology, oncology and pharmaceutical development will provide new perspectives in this field in the near future.

**Table 1:** Summary of various nanoadjuvants/delivery systems and their effects, safety aspects and clinical applications.

Type of Nanoadjuvant/Nanoparticulate Delivery System		Effect	Safety	Immune Target	Examples of Clinical Use	References
**Inorganic** **nanomaterials**	Nano-alum	NLRP3 inflammasome activation	Generally deemed safe Local adverse reactions (subcutaneous granuloma and other local inflammatory reactions)	Th1	/	[34,41,42,43]
	Silica nanoparticles	Delivery system with controlled antigen release	Safety is strongly dependent on preparation technique, physicochemical properties and administration route	Th1/Th2 Th17	/	[51,52,53,54,55,56,57,58]
	Clay nanoparticles	Depot effect	High immunogenicity; much higher compared to that of the FDA-approved adjuvants (e.g., Alum)	Th1	/	[64,67]
**Lipid-based** **nanoadjuvants**	Nanoemulsions	Sustained antigen release TLR4 ligand	O/W nanoemulsions (e.g., MF59^®^, AS03^®^ and AF03^®^) are less reactogenic and less toxic compared to W/O (e.g., Montanide^®^) nanoemulsions Narcolepsy (ASO3^®^ )	Th1/Th2	Vaccines against seasonal influenza, pandemic influenza and avian influenza; therapeutic vaccines against lung cancer and melanoma	[18,76,77,78,79,80,82,83,84,91]
	Lipid nanoparticles	Antigen delivery system (especially for mRNA) Antigen protection from degradation	Highly dependent on lipid components Local reactions and the injection site	Th1/Th2 CD8+ T cells	Vaccines against SARS-CoV-2	[107,108,109,169]
**Virus-based** **nanoparticles**	Adenoviral vector	Antigen delivery system	Local reactions and the injection site Fatigue, fever and headache Rare cases of thrombotic thrombocytopaenia	Th1/ Th2 CD8+ T cells	Vaccines against SARS-CoV-2 and Ebola	[118,121,129]
	Adeno-associated viral vector	Delivery system for antigens as well as for monoclonal antibodies and derivatives of immunoglobulins for passive immunization	Generally safe, but with low immunogenicity Transient and asymptomatic hepatitis	Th1/ Th2 CD8+ T cells	/	[118,133,134,170]
	Vesicular stomatitis viral vector	Delivery system for antigens	Fatigue, fever and headache	Th1/ Th2 CD8+ T cells	Vaccine against Ebola virus	[135,136,137,171]
	Lentiviral vector	Delivery system for antigens	Safety is still under evaluation	Th1/ Th2 CD8+ T cells	/	[138,172]
	Virus-like particles (VLPs)	Delivery system for antigens	Improved safety compared to viral-vectored vaccines Generally recognized as safe	Th1/ Th2 CD8+ T cells	Vaccines against hepatitis B virus, vaccines against human papilloma viruses (HPV), vaccine against hepatitis E virus and vaccine against malaria	[120,155,156]
	Virosomes	Delivery system for antigens	Improved safety profile compared to VLPs	Th1/Th2 CD8+ T cells	Vaccines against influenza and hepatitis A viruses	[167,168]

## Data Availability

Not applicable.
